# Transcriptome-Based Network Analysis Reveals Hirudin Potentiates Anti-Renal Fibrosis Efficacy in UUO Rats

**DOI:** 10.3389/fphar.2021.741801

**Published:** 2021-09-21

**Authors:** Hang-Xing Yu, Wei Lin, Kang Yang, Li-Juan Wei, Jun-Li Chen, Xin-Yue Liu, Ke Zhong, Xin Chen, Ming Pei, Hong-Tao Yang

**Affiliations:** ^1^ Department of Nephrology, First Teaching Hospital of Tianjin University of Traditional Chinese Medicine, Tianjin, China; ^2^ National Clinical Research Center for Chinese Medicine Acupuncture and Moxibustion, Tianjin, China; ^3^ Kidney Disease Treatment Center, The First Affiliated Hospital of Henan University of CM, Zhengzhou, China

**Keywords:** renal fibrosis (RF), natural products, hirudin (PubChem CID: 72941487), transcriptome (RNA-seq), UUO (unilateral ureteral obstruction)

## Abstract

**Background:** Hirudin has been widely used in the treatment of antifibrosis. Previous studies have shown that hirudin can effectively improve the clinical remission rate of chronic kidney disease. However, the mechanism of its renal protection has not been systematically investigated.

**Methods:** In this study, the reliability of UUO-induced renal interstitial fibrosis was evaluated by histopathological verification. High-throughput transcriptome sequencing was used to elucidate the molecular mechanism of hirudin, differentially expressed mRNAs were identified, and their functions were analyzed by GO analysis and GSEA. In addition, the RNA-seq results were validated by *in vitro* and vivo experiments.

**Results:** We found 322 identical differential expressed genes (IDEs) in the UUO hirudin-treated group compared with the sham group. Functional enrichment analysis indicated that cellular amino acid metabolic processes were the most obvious enrichment pathways in biological processes. In terms of molecular functional enrichment analysis, IDEs were mainly enriched in coenzyme binding, pyridoxal phosphate binding and other pathways. In addition, microbody is the most obvious pathway for cellular components. A total of 115 signaling pathways were enriched, and AMPK, JAK-STAT, and PI3K-Akt signaling pathways were the important signaling pathways enriched. We found that PI3K, p-Akt, and mTOR expression were significantly reduced by hirudin treatment. In particular, our results showed that hirudin could induce a decrease in the expression of autophagy-related proteins such as P62, LC3, Beclin-1 in TGF-β1-induced NRK-52E cells.

**Conclusion:** Our results suggest that hirudin may protect the kidney by ameliorating renal autophagy impairment through modulating the PI3K/Akt pathway.

## Introduction

Chronic kidney disease (CKD) is one of the major diseases that seriously endanger human health and is characterized by high incidence, low awareness, elevated medical costs and high risk of combined cardiovascular events (Yang C. et al., 2020; Luyckx et al., 2020). About 10.8% of adults in China have CKD, and the prevalence is as high as 18.3% in Central and Southwest China (Zhang et al., 2012; Huang et al., 2019). CKD affects 8–16% of the world’s population (Coresh, Josef et al., 2007; Jha, Vivekanand et al., 2013). In developed countries, CKD is most often attributed to diabetes and hypertension. However, less than 5% of people with early CKD report being aware of their disease (Chen et al., 2019). CKD usually has no obvious symptoms in the early stage, but when it progresses to the middle and late stage, as the kidney function declines, the body accumulates too many toxins leading to a series of uremic symptoms, requiring dialysis or even kidney transplantation, which seriously affects the quality of life of patients and brings a huge economic burden (Drawz and Rahman, 2015).

Renal interstitial fibrosis (RIF), a common pathway and the main pathological basis for the development of various CKD to end-stage renal disease (ESRD), mainly manifested as glomerulosclerosis and RIF, which is triggered by the inability of the kidney to be completely repaired due to abnormal tissue repair/regeneration after chronic and continuous injury, resulting in the replacement of normal tissue by fibrous tissue and loss of function, eventually causing renal failure, and the degree of RIF is closely related to the decline in renal function, often suggesting a poor prognosis for patients (Tang et al., 2019), is a common pathological feature of most CKD (Drawz and Rahman, 2015).

Therefore, to inhibit RIF is significant for the treatment of CKD. Many drugs used in clinical practice have anti-retrofibrotic effects, such as ACEI, ARB, aldosterone inhibitors, statins, endothelin receptors, beta-blockers, acetylsalicylic acid, metformin, and MMP inhibitors (Brewster and Perazella, 2004; Kanbay et al., 2009; Manns et al., 2012; Böhm et al., 2017; Rangarajan et al., 2018; Zhang et al., 2020), Among them, ACEI and ARB are the first-line drugs for the clinical treatment of CKD (KDOQI, 2012; Chen et al., 2018). The antifibrotic effect of ACEI/ARB may also arise from the preventive effect on glomerular injury induced by aldosterone or high intra-glomerular pressure (Obata et al., 1997; Wang et al., 2013). Although these drugs have shown some improvement in fibrosis, their therapeutic targets do not focus on fibrosis. Currently, the lack of exclusive drug targets for RIF and drugs targeting RIF severely hampers the treatment of CKD, so new drug targets and their targeting drugs are yet to be developed.

Hirudin is a bioactive protein extracted from the saliva of medical leeches, consisting of 65–66 amino acids, with a relative molecular weight of 7 kD, and is the main substance for leeches to exert their anticoagulant effect (Markwardt, 1991). Hirudin is excreted in urine principally in the form of prototype and metabolites through renal metabolism (Zhang and Lan, 2018). Studies have shown that hirudin is mostly distributed in the kidney and plasma, its pharmacokinetics is dose-dependent, and its metabolism *in vivo* depends on renal function (Robson et al., 2002)which provides a theoretical basis for the application of hirudin to blood concentrations in CKD.

Recently, hirudin has been used to treat diabetic nephropathy, IgA nephropathy and other chronic kidney diseases (Deng et al., 2019; Han et al., 2020), however, its specific mechanism of action in patients with CKD is not unclear. In this study, the regulatory mechanism of hirudin against CKD was investigated by RNA sequencing, differentially expressed gene (DEG) identification and annotation, gene ontology (GO) function and Gene Set Enrichment Analysis (GSEA) enrichment.

## Materials and Methods

### Animal Experiments

Eighteen healthy male SD rats with body weight (200 ± 20 g), purchased from Beijing Viton Lever Laboratory Animal Co. The animal production license number is SCXK (Jing) 2016–0006, and the animal quality certificate is No.110011200110536318. After 1 week of adaptive feeding, rats were randomly divided into sham group, unilateral ureteral obstruction (UUO) group, and UUO hirudin treatment group, with six rats in each group. Except for the sham group, the rats in all groups underwent left unilateral ureteral obstruction according to Martínez’s method (Martínez-Klimova et al., 2019). The rats were fasted the night before surgery and the left unilateral ureteral obstruction was performed under aseptic conditions. We first injected 10% chloral hydrate (3 ml/kg) intraperitoneally, and after the rats were anesthetized, they were fixed in prone position, and a longitudinal incision of about 1.5 cm in length was made on the left side of the spine, about 0.5 cm below the lumbar point of the ribs, and the left ureter were isolated, ligated and disconnected layer by layer. The rats in the sham group were treated as the UUO group except that they were not ligated and the ureter was not disconnected. The corresponding drug was given to each group of rats on the same day after the operation. The dose of the drug administered was determined according to the animal/human surface area method of conversion and combined with the results of the previous experiments (Yang K. et al., 2020). UUO hirudin treatment group with a dose of 40 IU/kg/d by tail vein injection, and the UUO group as well as Sham group with stroke-physiological saline solution (SPSS) by tail vein injection too. This study was approved by the Animal Protection and Utilization Committee of the first teaching hospital of Tianjin university of traditional Chinese medicine (Tianjin, China), in accordance with our institutional regulations.

### Sample Collection

Rats were anesthetized by intraperitoneal injection of 10% chloral hydrate (3 ml/kg) after 14 days of continuous tail vein administration. Subsequently, the rats were executed by cervical dislocation and the left kidney was removed. we divided the left kidney of each rat in each group into two parts along the coronal plane of the longitudinal axis of the kidney. A fresh half of the left kidney from all six rats in each group was fixed in 4% paraformaldehyde at room temperature for 24 h, while the other half of the left kidney from each group was rapidly frozen in liquid nitrogen and stored at −80°C until use.

### Morphology Analysis

After the rats were executed, the left side of the obstructed kidney tissue was left, and the kidney was incised along the sagittal plane and fixed in 10% neutral formalin solution for 24 h, followed by gradient dehydration, hyalinization, paraffin immersion, paraffin bedding, hematoxylin and eosin (HE) staining. Histological sections of the kidney were dewaxed and dehydrated, after that stained with an acidic complex of hematoxylin and Ponceau red liquid dye, followed by direct staining of the histological sections. Then soaked in 1% phosphomolybdic acid solution and stained directly with aniline blue solution and 1% acetic acid. The observed collagen is represented as a blue area for the degree of fibrosis, and the interstitial collagen deposition in Masson staining sections was observed under optical microscope.

### Cell Culture

Normal rat kidney epithelial cell line (NRK-52E) was purchased from Pronosai Life Sciences Co. (Wuhan, China). Cells were cultured in 90% high glucose DMEM (Thermo Fisher Science, Inc., United States) and 10% fetal bovine serum (FBS) at 37°C and 5% CO^2^ concentration. Then, NRK-52E cells were treated with 5 ng/ml TGF-β1 for 48 h to establish an *in vitro* fibrosis model.

### Cell Viability Assay

NRK-52E cells in excellent growth condition were prepared in cell suspension, and the cell density was adjusted to 8×10^4^ cells/mL at 100 μL per well and inoculated in 96-well plates. The cells were divided into control group and experimental group, and the experimental group was added with different concentrations (2.5, 5,7.5,10,12.5.15,17.5, and 20 IU/ml) of hirudin (patent no. ZL03113566.8,100 IU/vial, Canton Xike Kang Biotechnology Co. Ltd.,Guangxi, China), while the control group was added with fresh medium, and three replicate wells were set up for each group. After incubation for 24 and 48 h, 10 μL of CCK-8 reagent (Bioworld, BD0079-1) was added to each well and incubated for another 4 h. The 96-well plates were placed under an enzyme marker to determine the OD value at 450 nm, and the cell viability was calculated based on the OD value to determine the appropriate concentration of hirudin to continue the experiment. All samples were assayed at least three times.

### Detection of Fibrosis-Associated Protein Expression in Kidney Tissues by Western Blot

Total protein was extracted from obstructed kidney tissues or cells using 2×SDS lysate, and the protein concentration was determined by BCA protein assay kit (Solarbio life sciences Co., Beijing, China); 10% sodium dodecyl sulfate-polyacrylamide gel electrophoresis (SDS-PAGE) gel was used for electrophoretic separation, and the separation was transferred to PVDF membrane (Pierce Corporation, United States). The PVDF membrane was immersed in QuickBlock™ Western blocking solution (Beyotime Biotechnology, P0252) for 30 min at room temperature then incubated overnight at 4°C with anti-FN(1:2000, Proteintech, 15613-1-AP), anti-Col1 (1:1,000,Abcam, ab184993), anti-Col3 (1:1,000,Abcam, ab7778), anti- E-Cad (1:5,000, Proteintech, 20874-1-AP) , anti- PI3K (1:1,000, CST, 4,249), anti- Akt (1:1,000, CST, 4,691), anti-p-Akt (1:2000, CST, 4,060), anti-mTOR (1:1,000, CST, 1983), anti-Beclin1 (1:2000, Bioworld, AP0768),anti-P62 (1:500, Wanleibio,WL02385)and anti-GADPH (1:10,000, Proteintech, 10494-1-AP) respectively. After washing the membrane 3 times with TBST, the membrane was incubated with HRP-conjugated AffiniPure goat anti-mouse IgG (1:10,000, Proteintech, SA00001-1) or anti-rabbit IgG (1:10,000, Proteintech, SA00001-2) for 1 h at room temperature and washed 3 times again with TBST. Lastly, the membranes were added with enhanced chemiluminescence system (ECL) detection kit (Boster, China), ImageJ software was used for grayscale analysis of the image bands, and GAPDH was used as an internal reference for semi-quantitative analysis of the target proteins.

### Real-Time PCR

Total real-time polymerase chain reaction RNA was extracted using Trizol reagent (Invitgen, Carlsad, CA, United States), and the concentration of RNA for each sample was measured using an ND5000 ultra-micro UV spectrophotometer and reverse transcribed using the All-in-One™ First-Strand cDNA Synthesis Kit (GeneCopoeia Cat. No. AORT-0050) for reverse transcription. Target genes were analyzed by RT-qPCR according to the manufacturer’s instructions (Applied Biosystems Inc., Foster City, CA, United States). GAPDH forward primer is 5′-TCC​TGC​ACC​ACC​AAC​TGC​TTA​G-3'; reverse primer is 5′-AGT​GGC​AGT​GAT​GGC​ATG​GAC​T-3'; FN primer forward is 5 ′-ATG​AGA​AGC​CTG​GAT​CCC​CT-3′ and the reverse is 5′-GGA​AGG​GTA​ACC​AGT​TGG​GG-3'; COL1 is forward:5′- GGG​CAA​CAG​CAG​ATT​CAC​CTA​CAC-3′ and reverse:5′-CAAGGAATGGCAGGCGAGATGG-3'; for COL3, forward:5′- AGT​GGC​CAT​AAT​GGG​GAA​CG-3′ and reverse:5′-CACCTTTGTCACCTCGTGGA-3'. GAPDH was used as an internal control. Expression differences were assessed by the 2^−ΔΔCT^ method.

### Immunohistochemical Staining

Similar to HE staining, kidney tissues were fixed in paraffin and next cut into paraffin-embedded kidney sections using a microtome. The tissue sections were then dewaxed, rehydrated and subjected to antigen retrieval in 0.01 M citrate buffer (pH 6.0). After washing twice with PBS (0.01 M, pH 7.4), sections were prepared for blocking with primary antibodies against *α*-SMA (1:1,500, Proteintech, 14395-1-AP) and PI3K (1:300, Proteintech, 20584-1-AP) and incubated overnight, followed by biotin labeled secondary antibodies. Bound antibodies were visualized with diaminobenzidine (DAB) staining and imaged (magnification, × 200) using a light microscope (TE 2000, Nikon, Japan). Brown granule staining in the cytoplasm and/or nucleus represents positive expression of *α*-SMA and PI3K.

### Cellular Immunofluorescence Staining

NRK-52E cells were crawled, then treated with TGF-β1 and hirudin for 24 h, fixed with 4% paraformaldehyde, followed by the addition of 0.1% Triton X-100 to break the membrane for 10 min, and blocked with 10% FBS to eliminate non-characteristic fluorescent color development. After treatment with rabbit anti-rat LC3 (1:300, Proteintech, 14600-1-AP) and Beclin-1 (1:100, Bioworld, AP0768), and DyLight 488/594-labeled secondary antibody, the nuclei were stained with DAPI and sealed with anti-quenching blocker, and finally placed under a fluorescent microscope and photographed. The nuclei were stained with DAPI and blocked with anti-quenching blocker. The expression levels of the corresponding proteins were expressed as the integrated absorbance values.

### Transcriptional and Bioinformatics Analysis

High quality RNA was used for library construction and high-throughput sequencing. The RNA sequencing libraries were performed using the NEBNext Ultra RNA Library Prep Kit according to the manufacturer’s protocol. The library was subsequently sequenced by CaptalBio technology Co. Ltd. (Beijing, China) on a Novaseq6000 platform (Illumina, Chicago, IL, United States). Transcriptome analysis was performed using the rat reference genome-based read mapping. Gene expression levels were estimated using Fragments Per kilobase of an exon model per Million mapped fragments (FPKM) values.

### Statistical Analysis

All experiments were performed at least three times. SPSS 26.0 and GraphPad prism8 software were used for statistical analysis of the data, and all data were expressed as mean ± standard deviation. One-way analysis of variance (ANOVA) was used for comparison between groups. Tukey’s test was used to compare multiple methods. *p* < 0.05 was considered significant.

## Results

### Hirudin Mitigated Renal Interstitial Fibrosis

**FIGURE 1:**
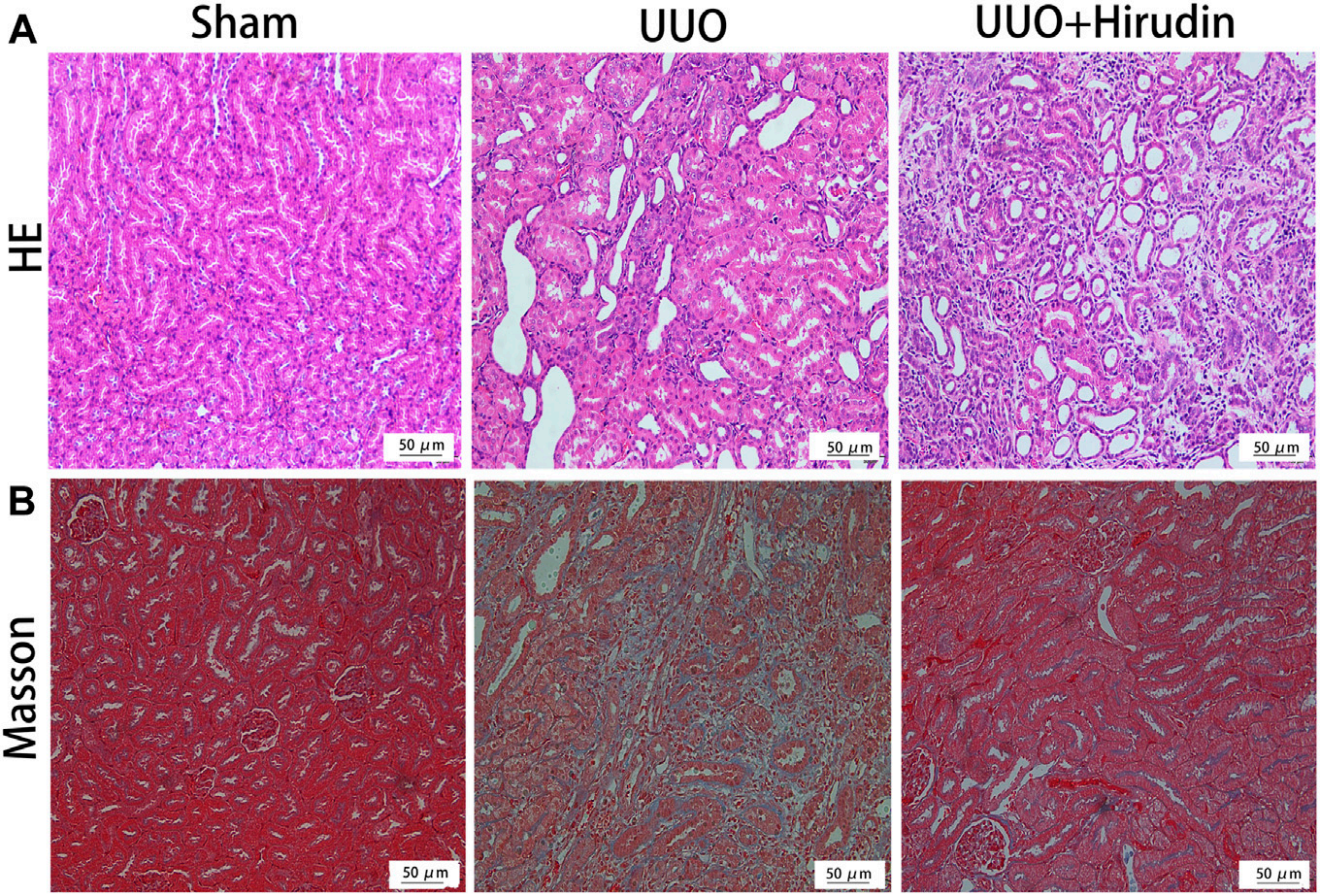
Effect of hirudin on renal histopathological alterations in unilateral ureteral obstruction (UUO)-induced renal interstitial fibrosis (RIF) rats. **(A)** HE staining showed that hirudin significantly reduced tubular dilatation or atrophy, interstitial fibrosis and inflammatory cell infiltration in the UUO rats (magnification×200). **(B)** Masson trichrome staining showed that hirudin significantly reduced renal tubulointerstitial damage and total collagen deposition in UUO rats. (magnification×400).

We initially investigated the effects of resveratrol on interstitial fibrosis in the kidneys of UUO models, which is a typical model of RIF (Song et al., 2014). There were no rats died during the experiment. The extent of kidney injury in the 14 days rats was evaluated by HE staining (Figure 1A). Briefly, the kidney tissue structure of the control group was normal. On the 14 days, the renal tubular structure of the UUO group rats was severely damaged, with massive necrosis of epithelial cells, atrophy and collapse of interstitium, thickening or disappearance of basement membrane, and obvious proliferation of renal interstitial fibrous tissue with a large number of monocytes and macrophages infiltration. The above pathological damage was significantly reduced after hirudin treatment compared with the UUO group. Masson trichrome staining was performed at 14 days to assess the extent of fibrosis in the rat kidney tissue (Figure 1B). No significant fibrosis was seen in the kidney tissues of the control rats. The model kidney disturbed cell arrangement, disrupted tubular structure and obvious proliferation of fibrous connective tissue; compared with the UUO group, the kidney fibrosis was reduced in the hirudin-treated group.

### Expressions of Fibrosis-Associated Genes in Rat Kidney Tissues

**FIGURE 2:**
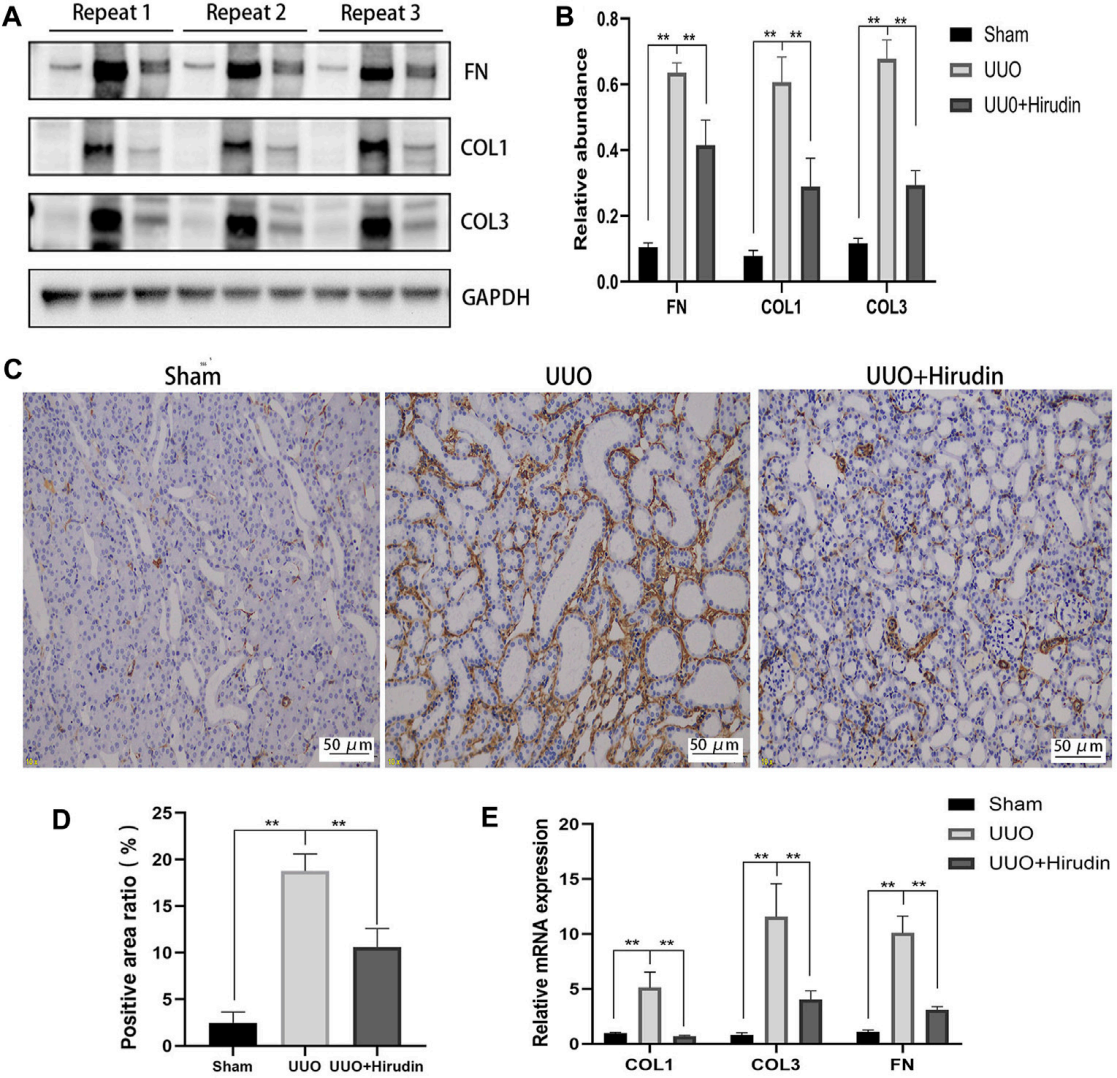
Effect of hirudin on inhibition of FN, COL1, COL3 and *α*-SMA induced by unilateral ureteral obstruction (UUO). **(A)** Effect of hirudin on FN, COL1 and COL3 in UUO kidney tissues, as determined by Western blot. **(B)** Effect of hirudin on FN, COL1 and COL3 in UUO kidney tissues, according to the results of Western blot. **(C)** Effect of hirudin on *α*-SMA in UUO kidney tissues, as determined by Immunohistochemical staining (magnification ×200). **(D)** Effect of hirudin on *α*-SMA in UUO kidney tissues, according to the results of Immunohistochemical staining. **(E)** Effect of hirudin on FN, COL1 and COL3 in UUO kidney tissues, according to the results of RT-qPCR. Data are expressed as mean ± SD for six rats per group. ^∗∗^ indicates *p* < 0.01and ^∗^ indicates *p* < 0.05.

Next, we examined the protein levels of fibrosis markers in the kidney tissues of each group. The expression of proteins COL1, COL3, and FN, which are closely related to fibrosis formation, was upregulated in the UUO group compared with the Sham group, and the expression was reversed after hirudin treatment (Figures 2A,B). Based on the biological importance of α-SMA in mediating RIF, we next assessed the protein levels of α-SMA in rat kidney tissues by immunohistochemistry (IHC). Compared with the Sham group, α-SMA positive expression was increased in the UUO group rats and was downregulated by hirudin treatment (Figures 2C,D). The expression trends of COL1, COL3, and FN mRNA were further confirmed by RT-qPCR to be consistent with protein expression (Figure 2E), and thus the transcript levels were statistically significant between the groups.

### Transcriptomic Analysis of Hirudin in the Treatment of Renal Interstitial Fibrosis

**FIGURE 3:**
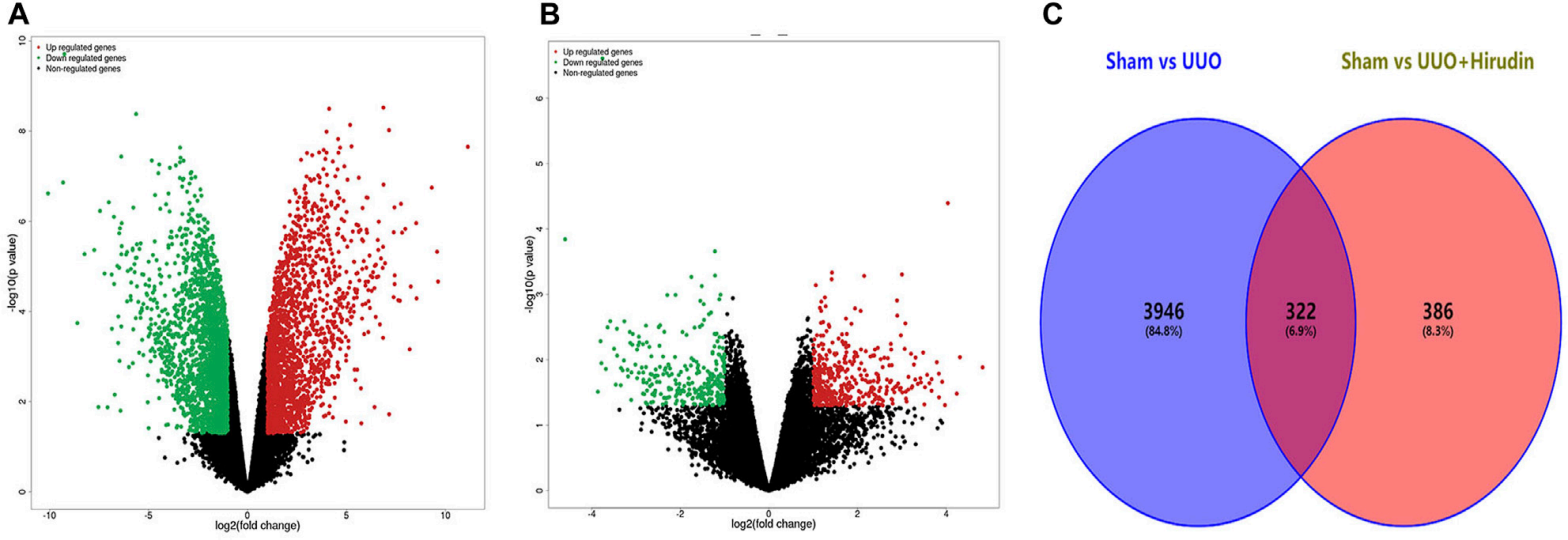
Transcriptomic assay and bioinformatics analysis. **(A)** Compared Sham group with UUO group, filtering of DEGs was presented by volcano. **(B)** Compared UUO hirudin-treated group with UUO group, filtering of DEGs was presented by volcano. **(C)** Venn diagram of overlapping genes derived from transcriptome analysis in a pairwise comparison. Red color represents upregulated genes, while green color shows downregulated genes.

We performed a comparative analysis of transcriptome analysis and gene expression between the Sham group, the UUO group and the UUO hirudin-treated group. In total, the RNA-Seq results included 30,408 differential expressed genes (DEGs, | log2FoldChange | ≥1). Among them, the Sham and UUO groups had 15,112 DEGs, while there were 15,296 DEGs between Sham and UUO hirudin-treated group. In which, 2013 genes were up-regulated and 2,255 genes were down-regulated in the UUO group compared to the Sham group (Figure 3A). 708 genes were changed, of which 419 genes were up-regulated and 289 genes were down-regulated in the UUO hirudin-treated group compared with the UUO group (Figure 3B). We then mapped the differentially expressed genes from the above two results to obtain 322 identical differential expressed genes (IDEs), which were the potential target genes for hirudin against kidney fibrosis (Figure 3C). Of these genes, 169 common genes were consistently upregulated and 153 common genes were consistently downregulated.

### PI3K/Akt Signaling Serves as a Candidate Pathway in Hirudin Against CKD

**FIGURE 4:**
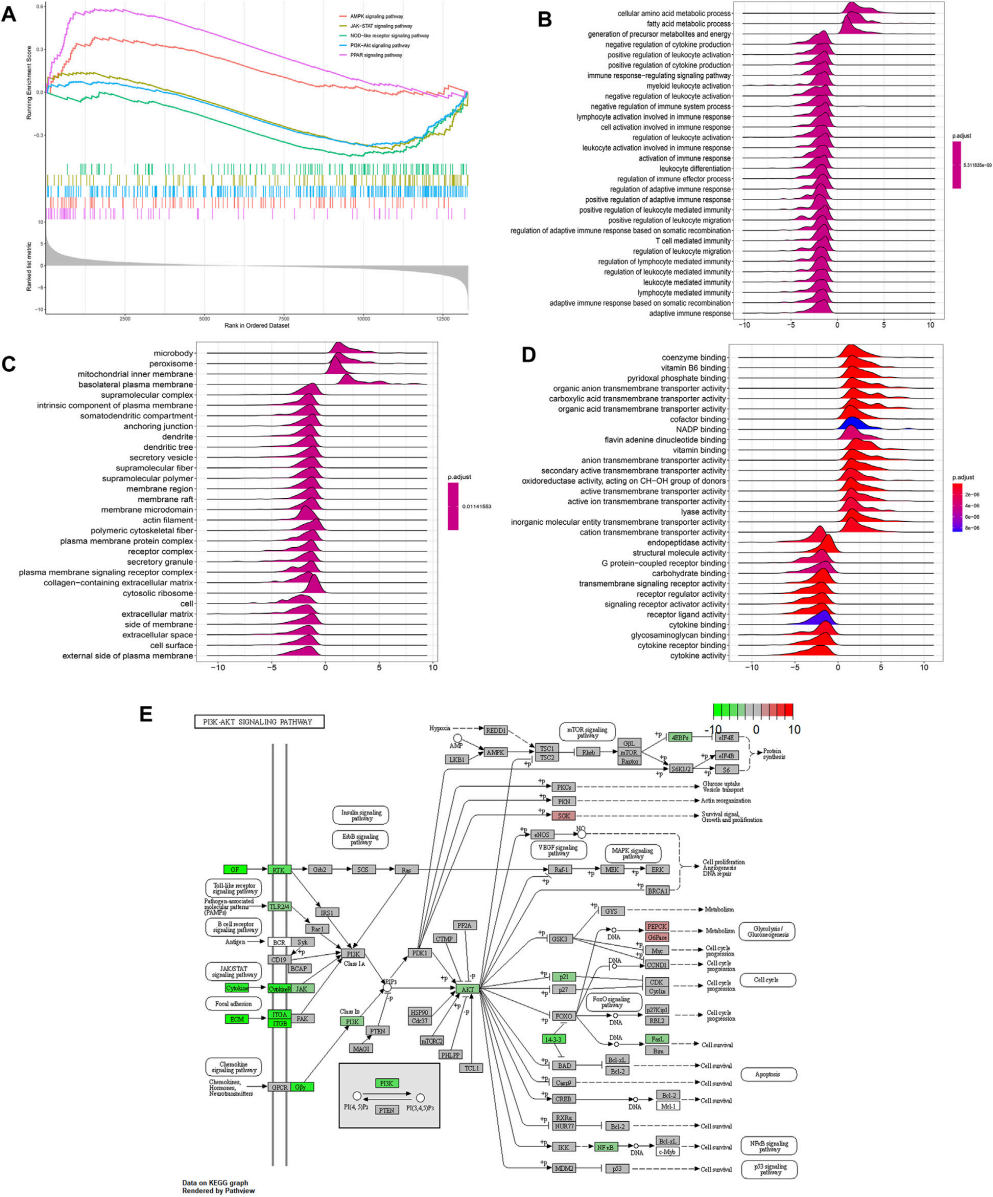
Functional analysis of the kidney transcriptome by hirudin treatment. **(A)** Gene Set Enrichment Analysis (GSEA) of hirudin treatment-related key pathways. **(B)** biological processes (BP) of GO analysis of hirudin treatment-related functional annotations. **(C)** molecular function (MF) of GO analysis of hirudin treatment-related functional annotations. **(D)** cellular component (CC) of GO analysis of hirudin treatment-related functional annotations. **(E)** Schematic diagram of hirudin against RIF through PI3K/Akt pathway.

To further discover the potential functional pathways of hirudin for CKD treatment, we performed GSEA analysis on IDEs. A total of 115 signaling pathways were enriched, as shown in Figure 4A, AMPK, JAK-STAT and PI3K-Akt signaling pathways were the important signaling pathways enriched. PI3K/Akt signaling pathway ranking five was priorly chosen to be verified in the following experiments. To explore the biological functions of IDEs, we performed Gene Ontology (GO) pathway annotation, and the results showed that cellular amino acid metabolic process was the most significantly enriched pathway in biological processes (Figure 4B). For molecular functional enrichment analysis, IDEs were mainly enriched in coenzyme binding, pyridoxal phosphate binding and other pathways (Figure 4C). In addition, microbody is the most obvious pathway for cellular components (Figure 4D).

### PI3K and Akt Play an Important Role in the Treatment of Chronic Kidney Disease With Hirudin

**FIGURE 5:**
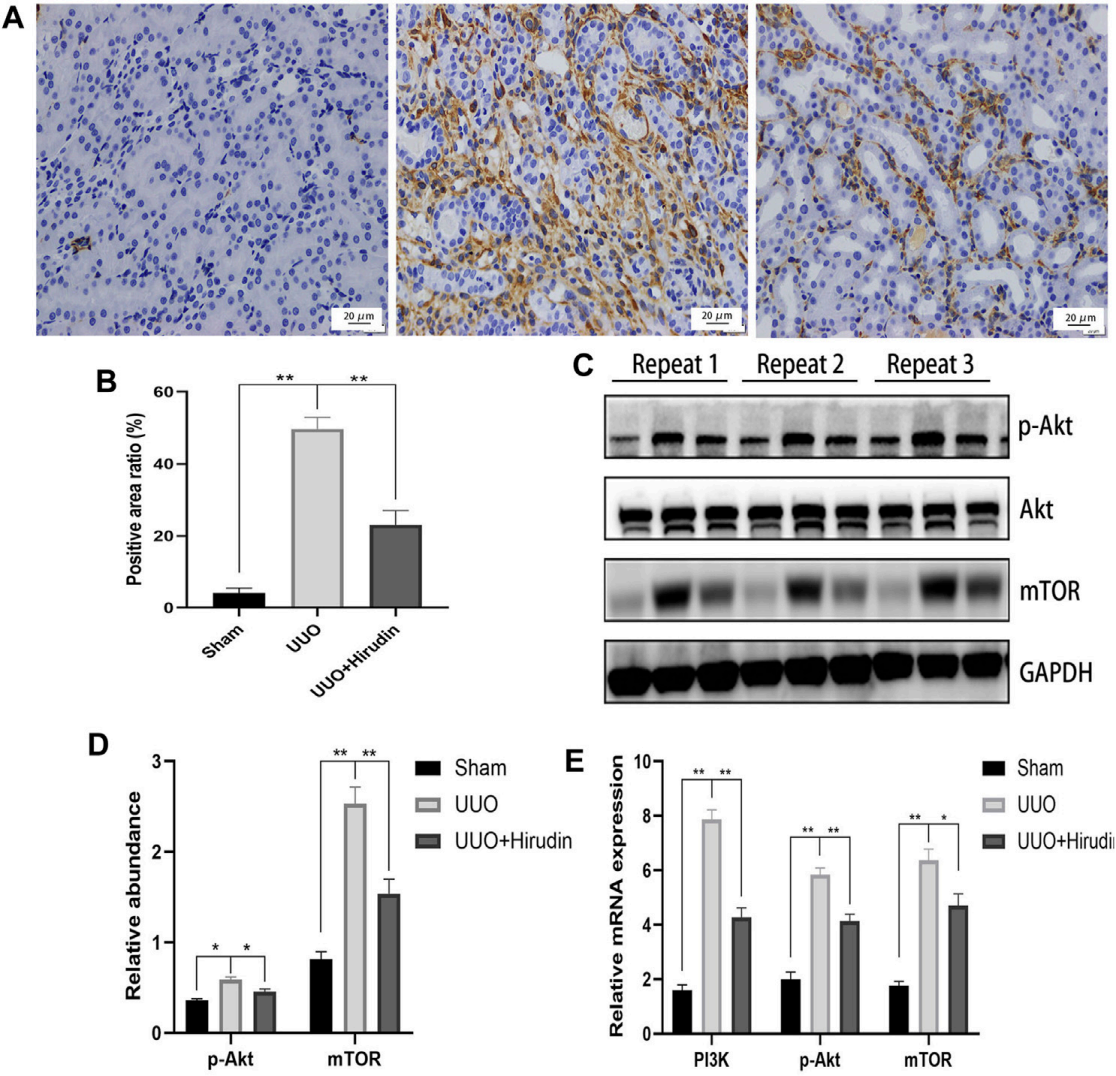
Hirudin inhibited the PI3K/Akt signaling pathway in UUO kidneys. **(A)** Effect of hirudin on PI3K in UUO kidney tissues, as determined by immunohistochemical staining. (magnification ×400) **(B)** Hirudin reduced the PI3K positive expression area in UUO kidneys, according to the results of immunohistochemical staining. **(C)** Effect of hirudin on p-Akt and mTOR in UUO kidney tissues, as determined by Western blot. **(D)** Effect of hirudin on p-Akt and mTOR in UUO kidney tissues, according to the results of Western blot. **(E)** Effect of hirudin on PI3K, p-Akt and mTOR in UUO kidney tissues, according to the results of RT-qPCR. Data are expressed as mean ± SD for six rats per group. ^∗∗^ indicates *p* < 0.01and ^∗^ indicates *p* < 0.05.

Based on the above results, PI3K, Akt, mTOR were selected as potential targets and their expressions were further examined by RT-qPCR. Figure 5E shows the mRNA expression trends of PI3K, p-Akt, and mTOR, and thus the transcript levels were statistically significant between the groups. In addition, using the same remaining samples for Western blot analysis, the expression of PI3K, p-Akt, and mTOR was significantly elevated in the UUO group of rats, whereas it was significantly suppressed in the UUO hirudin-treated group (Figures 5A–D). These results suggest that hirudin can inhibit the PI3K/Akt signaling pathway, which is involved in the construction of the UUO rats and hirudin treatment of RIF.

### Effect of Hirudin on NRK-52E Cell Viability After TGF-β1treatment

**FIGURE 6:**
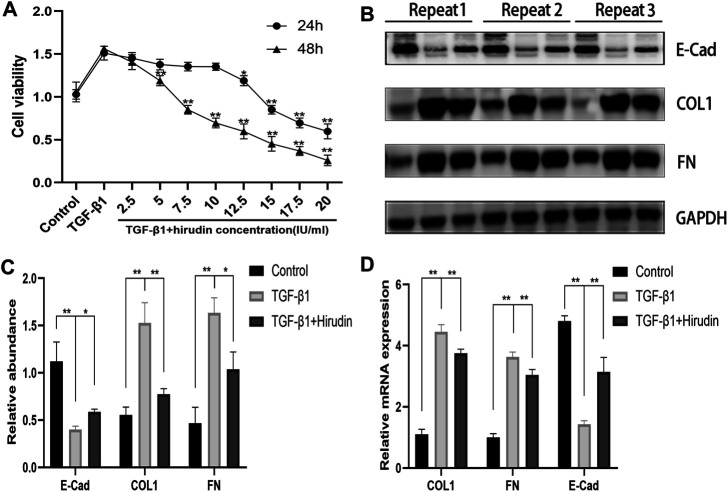
Hirudin attenuates renal interstitial fibrosis in TGF-β1 induced NRK-52E cells. **(A)** CCK-8 assay showed that the ability of cell viability was decreased in NRK-52E cells by treatment with hirudin. **(B)** Effect of hirudin on COL1, FN and E-Cad in NRK-52E cells, as determined by Western blot. **(C)** Effect of hirudin on COL1, FN and E-Cad in NRK-52E cells, according to the results of Western blot. **(D)** Effect of hirudin on COL1, FN and E-Cad in NRK-52E cells, according to the results of RT-qPCR. Data are expressed as mean ± SD (*n* = 3 for each group). ∗∗ indicates *p* < 0.01 and ∗ indicates *p* < 0.05.

To avoid the potential toxic effects of hirudin on NRK-52E cells and to explore the effective and safe dose of hirudin, we treated NRK-52E cells at 0,2.5,5,7.5,10,12.5,15, 17.5 and 20 IU/ml concentrations of hirudin for 24 and 48 h, respectively. Hirudin was found to significantly reduce the increase in cell viability induced by 10 ng/ml TGF-β1, and this inhibition showed a dose-dependent effect. In addition, cell viability was significantly inhibited at hirudin concentrations >10 IU/ml for 24 h and at hirudin concentrations >5 IU/ml for 48 h (Figure 6A). Therefore, we concluded that intervention with hirudin had no significant inhibitory effect on the cell viability of NRK-52E cells at a concentration of 10 IU/ml for 24 h. Thereby, we used 10 IU/ml of hirudin in the following experiments. To assess whether hirudin has antifibrotic properties *in vitro*, we analyzed the expression of fibrosis-associated proteins, which are reliable markers of TGF-β1-induced fibrosis after activation. Treatment with TGF-β1 significantly induced the expression of COL1, FN but decreased E-Cad expression.

We observed that treatment of NRK-52E with 10 IU/ml of hirudin for 24 h had an inhibitory effect on COL1 and FN expression in TGF-β1-induced NRK-52E cells, while promoting E-Cad expression (Figures 6B,C). This result was consistent with the expression of COL1, FN, and E-Cad at the mRNA level (Figure 6D).

### Hirudin Inhibits PI3K/Akt Signaling Pathway

**FIGURE 7:**
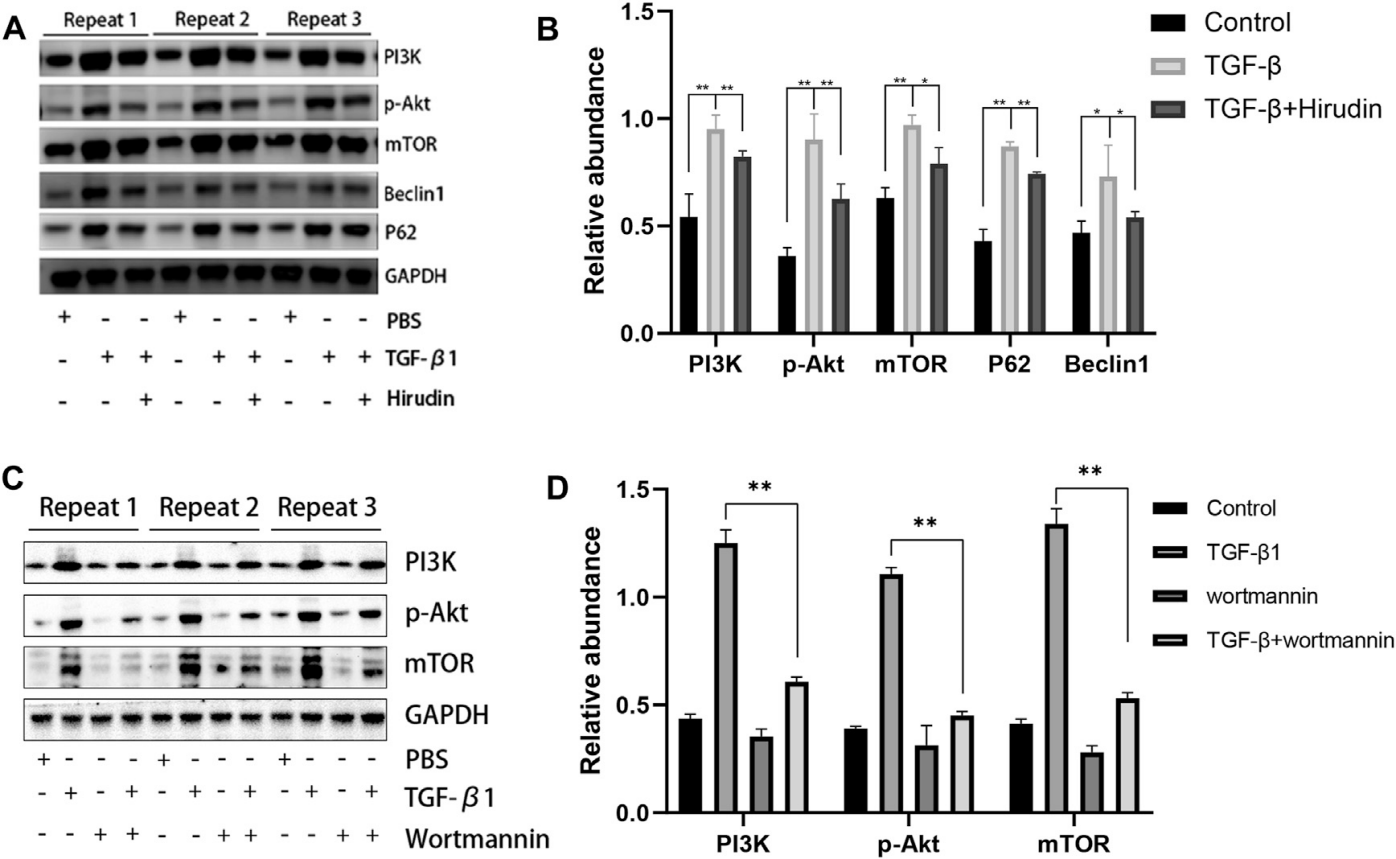
Hirudin inhibits the PI3K/Akt signaling pathway in TGF-β1-treated NRK-52E cells. **(A)** Effect of hirudin on PI3K, p-Akt, mTOR, Beclin1 and P62 in TGF-β1-treated NRK-52E cells, as determined by Western blot. **(B)** Effect of hirudin on PI3K, p-Akt, mTOR, Beclin1 and P62 in TGF-β1-treated NRK-52E cells, according to the results of Western blot. **(C)** Effect of wortmannin on PI3K, p-Akt, mTOR in TGF-β1-treated NRK-52E cells, as determined by Western blot.**(D)** Effect of wortmannin on PI3K, p-Akt, mTOR in TGF-β1-treated NRK-52E cells, according to the results of Western blot. Data are expressed as mean ± SD (*n* = 3 for each group). ∗∗ indicates *p* < 0.01 and ∗ indicates *p* < 0.05.

We further examined the effect of hirudin on regulating the PI3K/Akt pathway in NRK-52E. The expression of PI3K, p-Akt and mTOR was significantly upregulated after TGF-β1 treatment compared to the control group. However, hirudin treatment inhibited their increased expression (Figures 7A,B). Furthermore, we treated NRK-52E cells in the TGF-β1 group using the PI3K inhibitor wortmannin to investigate whether attenuating renal fibrosis in the TGF-β1 group was caused by inhibition of the PI3K/Akt pathway. As shown in Figures 7C,D, wortmannin treatment partially reversed the TGF-β1-induced increase in PI3K compared to cells in the TGF-β1 group. In addition, p-Akt and mTOR were correspondingly reduced by wortmannin treatment (*p* < 0.01). These data confirm that renal fibrosis is inhibited and restored by blocking the PI3K/Akt pathway.

### Hirudin Ameliorated Autophagy Disorders

**FIGURE 8:**
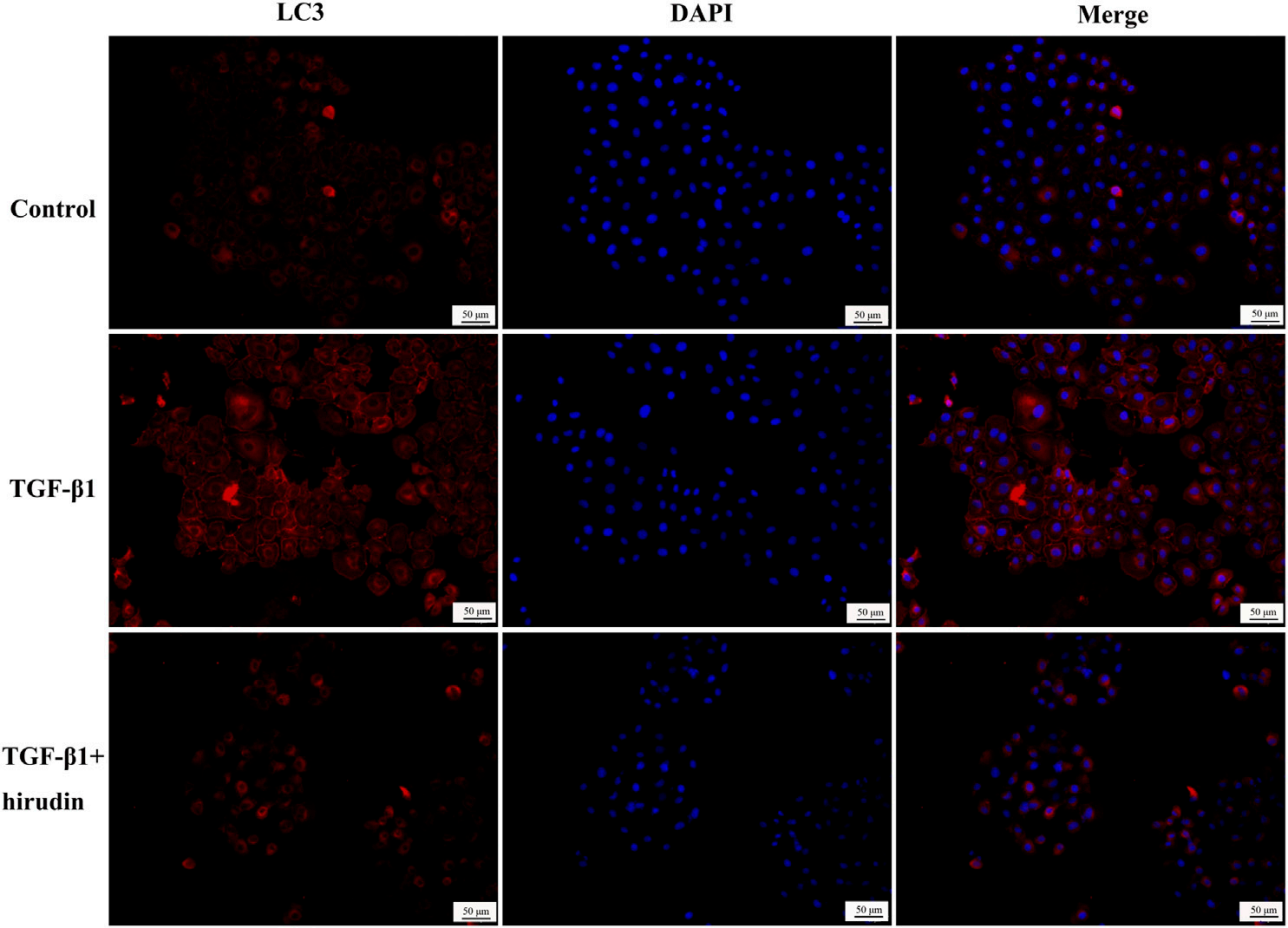
Cellular immunofluorescence detection of LC3 protein expression.

**FIGURE 9:**
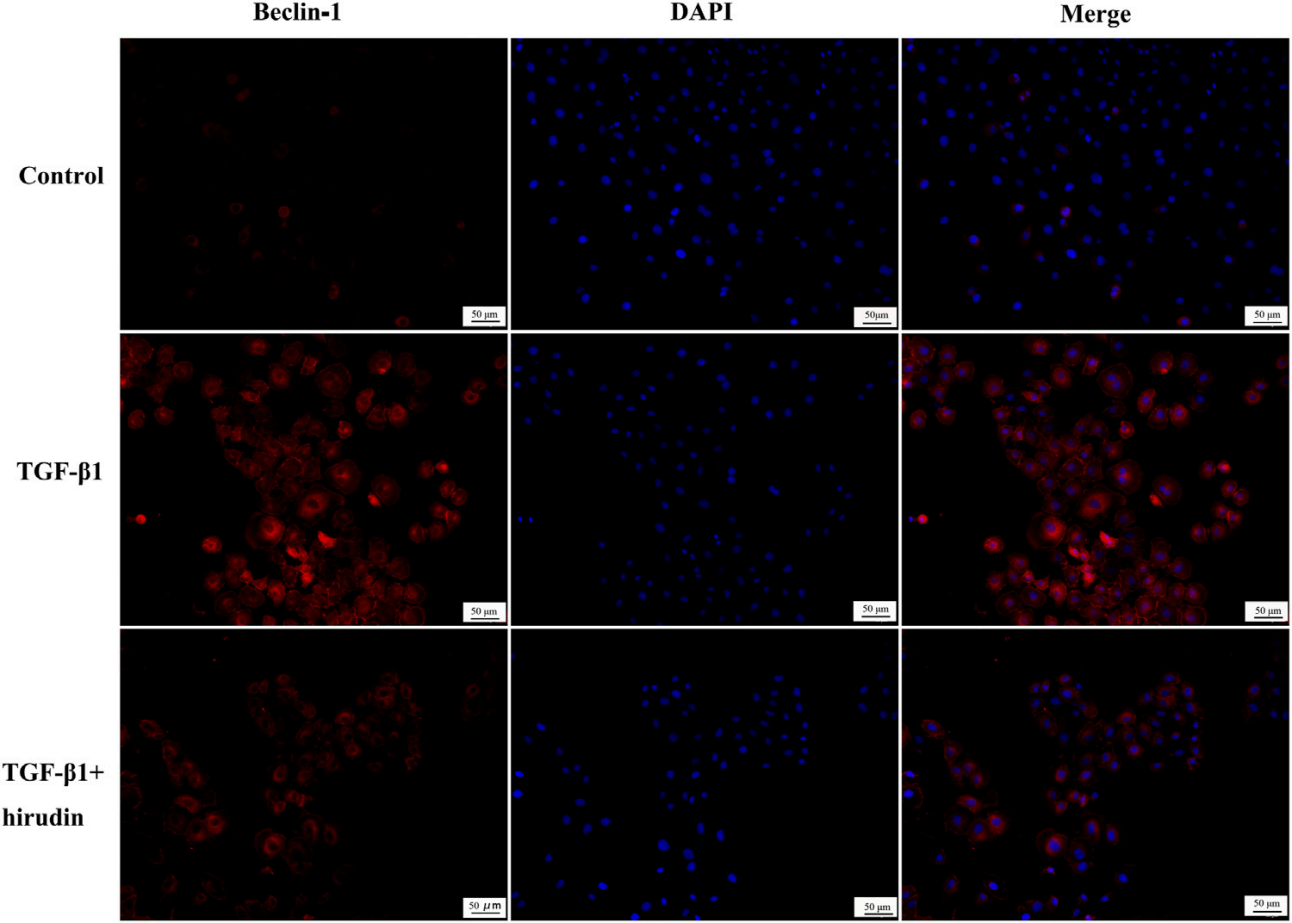
Cellular immunofluorescence detection of Beclin-1 protein expression.

Changes in autophagy are important for the physiological function of the kidney and the course of disease development (Kaushal et al., 2020; Tang et al., 2020). And PI3K/Akt signaling pathway is the classical pathway of autophagy (Reedquist et al., 2006; Jung et al., 2010). Therefore we then tested the effect of TGF-β1 on the autophagic capacity of NRK-52E cells. Western blotting results showed that the expression of P62 and Beclin-1 was increased in the TGF-β1 group compared with the Control group; the expression of P62 and Beclin-1 was significantly decreased in the TGF-β1+Hirudin group compared with the TGF-β1 group (Figures 7A,B). Immunofluorescence assay results showed that intracellular autophagy marker proteins LC3 and Beclin-1 were significantly increased in the TGF-β1 group compared with the Control group; intracellular LC3 and Beclin-1 were significantly decreased in the TGF-β1+Hirudin group compared with the TGF-β1 group (Figures 8, 9). In conclusion, hirudin could attenuate TGF-β1-induced autophagy impairment in NRK-52E cells.

## Discussions

Leeches are the dried bodies of the annelids W. pigra Whitman, H. nipponica Whitman, or W. acranulata Whitman, and modern pharmacological studies indicate that leeches mainly have anticoagulant and antithrombotic effects (Eldor et al., 1996). The hirudin in leeches is the strongest specific inhibitor of thrombin found so far (Markwardt, 1994). Therefore, the presence of hirudin in leeches and its content has become an important criterion for measuring the efficacy of medicinal leeches (Zhang et al., 2013). In addition to its anticoagulant effect, hirudin also has some anti-inflammatory and anti-fibrotic effects (Li et al., 2019; Shen et al., 2019). However, there are few reports on the anti-nephrogenic fibrotic effects of hirudin. UUO is a common way to elucidate the pathological mechanisms associated with RIF, such as glomerulosclerosis, inflammatory cell infiltration, interstitial ECM aggregation, and collagen deposition (Nishida et al., 2007; Islam et al., 2016). In this study, we evaluated the effect of hirudin in improving renal function by detecting renal fibrosis indexes. The experimental results illustrated that hirudin significantly improved the renal fibrosis-related protein levels in UUO rats, suggesting its protective effect on renal function in UUO rats. To further investigate the molecular mechanism of the renal protective effect of hirudin, we identified 322 mRNAs (including 169 up-regulated mRNAs and 153 down-regulated mRNAs) that were differentially expressed in the hirudin-treated group compared with the UUO group.

Functional enrichment analysis showed that these genes were significantly enriched in relation to Go-BP, such as negative regulation of cytokine production, positive regulation of cytokine production, adaptive immune response, activation of immune response and cell activation involved in immune response, were significantly enriched. response, activation of immune response and cell activation involved in immune response, etc. It was shown that cytokines TGF-β, HIF-1, and NLRP3 promote the development of chronic kidney disease and influence the immune status of CKD. Corresponding to the GO results, pathway enrichment analysis enriched 115 terms, including AMPK signaling pathway, JAK-STAT signaling pathway and PI3K-Akt signaling pathway.

The adenylate-activated protein kinase (AMPK) pathway is involved in the development of CKD (Guo et al., 2014; Ha et al., 2014). Activation of the AMPK signaling pathway reduces oxidative stress in chronic kidney disease (Kidokoro et al., 2013), decreases the expression of inflammatory factors in plasma and renal tissues of CKD patients (Xie et al., 2017), and inhibits the development of renal tubular fibrosis (Ishibashi et al., 2012).

The JAK/STAT signaling pathway is an important class of cytokine signaling pathway that is widely involved in cell proliferation, differentiation, apoptosis and inflammatory response, and has a regulatory role in various renal diseases (Chuang and He, 2010; Wiezel et al., 2014; Pace et al., 2019). This pathway can be involved in the development of obstructive nephropathy, diabetic nephropathy and acute kidney injury by regulating the expression of JAK and STAT family factors, and inhibition of this signaling pathway can help to slow down the progression of CKD (Liu et al., 2020; Zhao et al., 2020).

The phosphatidylinositol kinase-3 (PI3K)/protein kinase B (Akt) signaling pathway is also an important signaling pathway in chronic kidney disease, and overactivation of this signaling pathway can trigger the onset and development of CKD (Lu et al., 2019). We randomly selected the PI3K-Akt signaling pathway for validation. Our study suggests that PIK3/Akt signaling pathway activation is involved in the development of CKD. After hirudin treatment, PI3K protein was reduced in renal tissues of UUO rats as well as in TGF-β-induced NRK-52E cells. The same trend was observed for Akt phosphorylation levels. We conclude that hirudin is able to alleviate renal tissue injury by inhibiting the activation of the PIK3/Akt signaling pathway. The PI3K/Akt signaling pathway is the most classical signaling pathway in the regulation of cellular autophagy, and it plays an important role (Chen and Debnath, 2013; Yu et al., 2015). In recent years, an increasing number of studies have found that PI3K/Akt pathway-regulated cellular autophagy is closely associated with the development and pathological progression of chronic kidney diseases such as diabetic nephropathy and RIF (Kimura et al., 2017; Liu et al., 2017; Lin et al., 2019).

There is also extensive research evidence further suggesting the presence of autophagy-deficient kidneys in CKD patients (Kume et al., 2012; Lin et al., 2019; Choi, 2020). Based on these findings, the hypothesis that defective autophagy in the kidney may increase the susceptibility of renal cells to the associated injury, leading to treatment-emergent resistant proteinuria and progressive decline in renal function, was then proposed (Leventhal et al., 2014). Therefore, restoring autophagy as a treatment for CKD may become a new therapeutic option. LC3 is widely used as a marker of autophagy, and the transition from LC3I to LC3II suggests the formation of autophagosomes. In addition, ubiquitin-binding protein P62 is another marker protein reflecting autophagic activity, and its accumulation in the cytoplasm suggests diminished autophagic activity. Beclin1 also positively regulates autophagic activity by binding to PI3K3C, forming a core complex that initiates autophagy (Yang and Klionsky, 2010). Our results showed that the expression levels of P62, Beclin-1 and LC3 were significantly increased after TGF-β intervention in NRK-52E cells, suggesting an intracellular impairment of autophagy, which was reversed by hirudin. Therefore, we suggest that hirudin has a nephroprotective effect and ameliorates autophagy impairment in renal injury, and this effect is associated with the PIK3/Akt signaling cascade.

In this study, we investigated the mechanism of action of hirudin against RIF from the perspective of transcriptomic analysis and *in vivo* and *in vitro* experimental validation, and there are some areas for improvement in the follow-up study. First, clinical data and samples need to be studied to support our findings. Second, RIF is a complex pathological process involving various mechanisms such as autophagy, oxidative stress, impaired energy metabolism, and apoptosis, and further elucidation is still needed for the specific mechanism of hirudin against RIF.

The anti-renal fibrotic effect of hirudin has been studied, but its mechanism of action has not been fully elucidated. In the present study, we investigated the molecular mechanism of the anti-renal fibrosis effect of hirudin from the whole transcriptome and *ex vivo* experiments. The results showed that hirudin ameliorated renal fibrosis in rats by a mechanism involving the regulation of the PIK3/Akt signaling pathway and thus the activation of autophagy.

## Data Availability

The data presented in the study are deposited in the GEO repository, accession number GSE181380.

## Abbreviations

α-SMA, α smooth muscle actin; ACEI, angiotensin converting enzyme inhibitors; Akt, protein kinase B; AMPK, Adenosine 5-monophosphate (AMP)-activated protein kinase; ANOVA , analysis of variance; ARB, Angiotensin Receptor Blocker; CKD, Chronic kidney disease; Col1, Recombinant Collagen Type I; DAB, diaminobenzidine; DEG, differentially expressed gene; E-Cad, E-Cadherin; ECL, enhanced chemiluminescence system; ECM, extracellular matrix; ESRD, end-stage renal disease; FBS, fetal bovine serum; FN, Fibronectin; FPKM, Fragments Per kilobase of an exon model per Million mapped fragments; GADPH,glyceraldehyde-3-phosphate dehydrogenase; GO, gene ontology; GSEA, Gene Set Enrichment Analysis; HE, hematoxylin and eosin ; HIF-1,Hypoxia-inducible factor 1; IgA, Immunoglobulin A; IDEs, identical differential expressed genes; IHC, immunohistochemistry; JAK-STAT, Janus kinase-signal transducer and activator of transcription; LC3, light chain 3; MMP, Mitochondrial membrane potential; mTOR, mammalian target of rapamycin; NLRP3, Nlrfamily pyrin domain-containing 3; NRK-52E, Normal rat kidney epithelial cell line; PI3K, phosphatidylinositol kinase-3; RIF, Renal interstitial fibrosis; SDS-PAGE, sodium dodecyl sulfate-polyacrylamide gel electrophoresis; SD rats, Sprague-Dawley rats; TGF-β1, transforming growth factor-β1; UUO, unilateral ureteral obstruction.
